# Changes in cannabis potency and cannabis-related psychiatric harms: a 23-year ecological study in Denmark

**DOI:** 10.1017/S0033291726104036

**Published:** 2026-05-04

**Authors:** Kristine Rømer Thomsen, Birgitte Thylstrup, Morten Hesse, Christian Lindholst, Lotte Ask Reitzel, Tina Stokholm Eriksen, Annette Erlangsen, Amir Englund, Tom P. Freeman, Carsten Hjorthøj

**Affiliations:** 1 Centre for Alcohol and Drug Research, Department of Psychology and Behavioural Sciences, Aarhus University, Denmark; 2 Department of Forensic Medicine, Aarhus University, Denmark; 3 Department of Forensic Medicine, University of Copenhagen, Denmark; 4 Department of Forensic Medicine, University of Southern Denmark, Denmark; 5 Copenhagen Research Centre for Mental Health, Mental health services in the Capital Region of Denmark, Denmark; 6 Department of Mental Health, Johns Hopkins Bloomberg School of Public Health, USA; 7 Centre for Mental Health Research, National Centre for Epidemiology and Population Health, The Australian National University, Australia; 8 Centre for Neuroimaging Sciences, Institute of Psychiatry, Psychology, and Neuroscience, King’s College London, UK; 9 Department of Psychology, University of Bath, UK; 10 Section of Epidemiology, Department of Public Health, University of Copenhagen, Denmark

**Keywords:** cannabis, cannabis use disorder, dual diagnosis, potency, psychosis, schizophrenia, ∆-9-tetrahydrocannabinol, cannabis resin

## Abstract

**Background:**

In recent decades, the potency of cannabis resin increased globally, raising concerns, as higher potency has been associated with increased risk of psychiatric harms at the individual level. The aim here was to examine whether changes over time in the potency of seized cannabis resin samples were associated with psychiatric harms at the population level.

**Methods:**

Data on ∆-9-tetrahydrocannabinol (THC) concentration in seized cannabis resin were obtained from forensic departments in Denmark (2000–2022), the country reporting the highest potency in Europe. Data on admissions to cannabis treatment, incidence of cannabis-induced psychosis, and dual diagnosis (schizophrenia and cannabis use disorder) were obtained from national registers. Time-dependent associations between potency and the outcomes were examined with mixed-effects linear regression models and associations across age and sex were explored. Candidate time lags were 0–10 years.

**Results:**

THC concentration increased almost fourfold: mean 8.3–31.2% from 2000 to 2022. In fully adjusted models, THC was positively associated with first-time cannabis treatment entry at lags of 0–6, strongest at year 0 (*p <* 0.0001); incidence of cannabis-induced psychosis at lags of 0–4, strongest at year 0 (*p* < 0.0001); and incidence of dual diagnosis at lags of 0–1, strongest at year 0 (*p <* 0.01). No positive associations were found in unadjusted models. Subgroup analyses indicated associations in older patients and women.

**Conclusions:**

Potency of seized cannabis resin increased almost fourfold from 2000 to 2022. Changes in cannabis potency were positively associated with psychiatric harms at the population level across all outcomes.

## Introduction

Cannabis is one of the most widely used substances globally (UNODC, 2023). Its potency is measured by the concentration of its primary psychoactive component, ∆-9-tetrahydrocannabinol (THC), which typically is captured as the combined amount of THC and THC acid (THCA). An increase in potency has been reported across the United States and Europe during the past two decades (Freeman et al., 2021). A particularly prominent example of this is found in Denmark, where THC concentration in seized cannabis resin (also referred to as hash or hashish) increased threefold from a mean of 8.3% in 2000 to 25.3% in 2017 (Rømer Thomsen et al., 2019).

The increased potency of cannabis raises concerns as high levels of THC have been associated with increased risk of adverse outcomes related to mental health. A recent review of observational studies found that potency was strongly associated with psychosis and cannabis use disorder, while evidence varied for anxiety and depression (Petrilli et al., 2022). These findings were, for instance, based on large case–control studies with data from several European countries and Brazil (Di Forti et al., 2019; Quattrone et al., 2021), which showed an increased risk of first-time psychosis in individuals reporting using higher potency forms of herbal cannabis (Di Forti et al., 2019). Similarly, a recent review of observational and experimental studies found stronger evidence for positive associations between potency and outcomes related to psychosis than for other psychiatric outcomes (Lake et al., 2025).

It is plausible that the increase in cannabis potency over time could be associated with an increase in the number of individuals with cannabis use disorder and psychosis at the population level. This has been examined in a limited number of studies to date. An observational study from the Netherlands found time-dependent associations between changes in THC concentration of herbal cannabis sold in retail outlets and first-time admissions to cannabis treatment during 2000–2015 (Freeman et al., 2018). More specifically, THC concentration was positively associated with treatment entry at lags of 5–7 years after adjusting for age, sex, and non-cannabis drug treatment admissions, with the strongest association at 5 years. Recently, this was corroborated in an ecological study from Germany showing that with an increase in the median THC concentration of seized cannabis flowers of one percentage point, the proportion of cannabis-related ICD-10 diagnoses increased by 0.17 percentage points for women and by 0.42 percentage points for men between 2009 and 2021. The strongest effect was at time lag 0 years, with a continuous decrease in the effect strength with increasing delay and no significant correlation after a time lag of 6 years (Manthey, Rosenkranz, Jonas, & Schwarzkopf, 2024).

While several laboratory studies have shown that administration of THC elicits acute dose-dependent intoxication, negative effects on cognitive functions, and anxiety and psychotic symptoms in infrequent users (Curran et al., 2002; D’Souza et al., 2004; Hindley et al., 2020; Morrison et al., 2009), the two studies above are the only studies to date examining links between potency and real-world evidence of cannabis-related psychiatric harms. Hence, more studies are needed to examine if changes in potency over time of popular cannabis products are linked with changes in risk of cannabis-related psychiatric harms at the population level. An important question is whether potency is linked with real-world evidence of cannabis use disorder and psychosis over time, and whether potential links with these harms are equally strong. Given the severe and debilitating nature of psychotic disorder, and that cannabis-induced psychosis often progresses to schizophrenia (Arendt et al., 2005; Murrie, Lappin, Large, & Sara, 2020), the link between cannabis potency and cannabis-induced psychoses is especially important.

Denmark is a particularly important country for undertaking these examinations, as Denmark reported the highest THC concentration in seized cannabis resin across Europe (Freeman et al., 2019), and the highest use of cannabis resin among 15-year-olds across the Nordic countries (ESPAD Group, 2020, 2025). In Denmark, cannabis resin is by far the most popular type of cannabis used for recreational purposes based on police seizures (Danish Health Authority, 2023a) and surveys on consumption patterns (Callesen et al., 2024; Frederiksen et al., 2025), and is the most frequently reported main drug in specialized drug treatment (Danish Health Authority, 2024a). Cannabis use has not been legalized or decriminalized, but in 2019, a Medical Cannabis Pilot Program was initiated, allowing prescription of a limited number of products to patients with multiple sclerosis, spinal cord injury, chronic pain, or chemotherapy-related nausea.

The aim of the present study was to examine whether changes over time in potency of seized cannabis resin samples in Denmark were associated with three key measures of cannabis-related psychiatric harms:

- Incidence rate of first-time admissions to specialized treatment for cannabis problems
- Incidence rate of cannabis-induced psychosis
- Incidence rate of dual diagnosis (schizophrenia and cannabis use disorder).

Additionally, we explored the roles of sex and age. Based on previous studies, we hypothesized that changes in THC concentration would be positively linked to the above measures of cannabis-related psychiatric harms.

## Methods

### Cannabis resin

Data on THC concentration (combined amount of THC and THC acid, THCA) in seized cannabis resin (also referred to as hash or hashish) samples from 2000 to 2022 were obtained from the three forensic departments, which serve all police districts in Denmark: Copenhagen (covering eastern Denmark), Aarhus (covering western Denmark), and Odense (covering southern Denmark). Not all cannabis seizures are subjected to forensic analyses, and the decision relies on a local police professional assessment in each case. Therefore, only a limited number of samples of the national seizures are analyzed and available as study material. However, because internal reports from forensic laboratories showed an increase in the THC concentration of cannabis resin in 2012 and 2013, the Danish Health Authority decided to include cannabis resin in the national monitoring program (*Drugs on Street Level*) from 2014. The period between 2000 and 2013, therefore, included cannabis resin samples analyzed by forensic laboratories from all police districts across Denmark, whereas those included in the period between 2014 and 2022 consisted of systematically collected resin samples from 3 of the 12 police districts: Copenhagen, Funen (including Odense), and Eastern Jutland (including Aarhus). Every month, the police in Copenhagen, Funen, and Eastern Jutland selected a small seizure of cannabis resin, which was sent for analysis in the associated forensic laboratory, amounting to 12 samples from each of the three areas per year.

Because samples from 2014 to 2022 all stem from the police districts of Copenhagen, Funen (including Odense), and Eastern Jutland (including Aarhus), we opted to restrict the analyses of admissions to treatment for cannabis problems, incidence of psychosis, and incidence of dual diagnosis to those specific areas. The rationale was that people who used cannabis and lived within 40 kilometers of Copenhagen, Aarhus, and Odense were likely to have obtained their cannabis resin from these larger cities or other places within these police districts. Hence, we identified and included individuals living in municipalities in these areas in our analyses, while individuals living in other areas of Denmark were excluded (see Supplementary File 1 for more details).

#### Chemical analyses

The three forensic laboratories calculated the total THC concentration (percent of THC and THCA by weight of sample, expressed in THC equivalents) of cannabis resin. Chemical analysis was conducted using either gas chromatographic analysis with flame ionization detection (GC-FID) or liquid chromatographic analysis with mass spectrometric detection for the quantification of the total THC content. The conversion formula (total THC = THC + 0.877 × THCA) was applied for the calculation of results from HPLC analysis. All laboratories have been accredited according to the ISO 17025 standard by the Danish Accreditation Fund (DANAK). The analytical methods used for THC quantification have been validated and accredited since 2009 (Copenhagen lab) and 2011 (Aarhus lab). The method used in the regional laboratory in Odense was not validated.

### Cannabis treatment, cannabis-induced psychosis, and dual diagnosis

National data on admissions to specialized treatment for cannabis problems were obtained from the *Registry of Drug Users in Treatment* (Danish Health Authority, 2023b), the *National Patient Register* (Lynge, Sandegaard, & Rebolj, 2011), and the *Psychiatric Central Research Register* (Mors, Perto, & Mortensen, 2011) from 2000 to 2022. The *Registry of Drug Users in Treatment* contains records of admissions to Danish community outpatient treatment for drug problems since 1996 and delivers monitoring data to the European Union Drug Agency (EUDA) regarding treatment demand indicators (Montanari et al., 2019). We obtained data on the main drug, which was self-reported by users. The *Psychiatric Central Research Register* has listed records of all inpatients treated at psychiatric departments in Denmark since 1969, as well as outpatients (including emergency departments) treated since 1995. Similarly, the *National Patient Register* contains data on all inpatients treated in somatic hospitals from 1977 and all outpatients seen from 1995. Admissions to specialized treatment for non-cannabis drug problems were used to adjust for systemic factors that may impact admissions to specialized treatment for drug problems in general.

Data on the incidence of cannabis-induced psychosis and the incidence of dual diagnosis, defined as the co-occurrence of schizophrenia and cannabis use disorder, were obtained from the national *Psychiatric Central Research Register* (Mors et al., 2011) from 2000 to 2022. For outcome two, we used information on cannabis-induced psychosis (ICD-10 code F12.5), and for outcome three, we used information on cannabis harmful use or dependence (F121 and F122) and schizophrenia (F20). Other types of substance-induced psychosis (F1X.5) were used to adjust for systemic factors that may impact the diagnostic evaluation of substance-induced psychosis in general (outcome two). Similarly, other types of substance use disorders (F1X.1 and F1X.2, except F12.1 and F12.2) were used to adjust for systemic factors that may impact the diagnostic evaluation of substance use disorders in general.

For all three outcomes (specialized treatment for cannabis problems, cannabis-induced psychosis, and dual diagnosis), we obtained data from citizens living in regions overlapping with police seizures, as described above.

### Statistical analyses

We used mixed-effects linear regression models to examine time-dependent associations between THC concentration in cannabis resin samples and the incidence rate of first-time cannabis admissions to specialist treatment for cannabis problems, cannabis-induced psychosis, and dual diagnosis. Consistent with previous research (Freeman et al., 2018), we adjusted for age, sex, and admission to specialized treatment for non-cannabis drug problems/other substance-induced psychosis/other substance use disorder, and we estimated associations at candidate time lags of 0–10 years. Additionally, findings were stratified by sex and age. Findings were considered significant at *p <* 0.01.

Danish law does not require an ethics evaluation or informed consent for the types of data used in this study (including register) to be used for research if data are used solely for statistical and scientific purposes (Thygesen, Daasnes, Thaulow, & Brønnum-Hansen, 2011).

## Results

### Changes in potency of seized cannabis resin from 2000 to 2022

THC concentration was available from 610 unique cannabis resin samples from 2000 to 2022.

**Figure 1.**
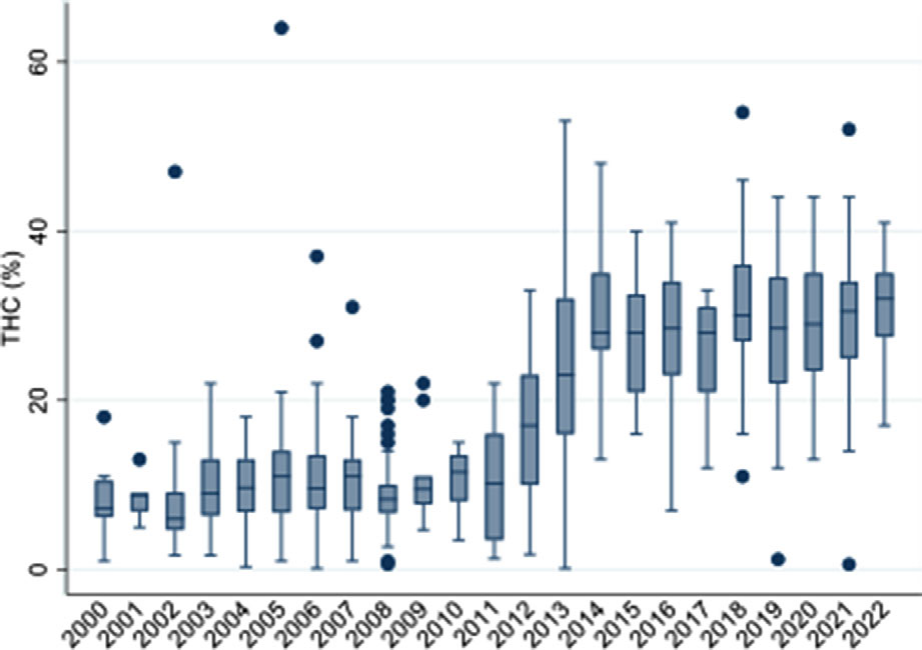
THC concentration in individual seizures by year.

In these samples, the THC concentration increased almost fourfold from a mean of 8.3–31.2% (see Figure 1). The largest increase was observed between 2011 and 2014, where the median potency increased from 10.2 to 28.0% in just 3 years.

### Time-dependent associations between potency and cannabis treatment

We did not find significant associations between THC concentration and first-time cannabis treatment when assessed in crude and in sex- and age-adjusted models. When additionally adjusting for non-cannabis drug treatment, THC concentration was positively associated with cannabis treatment at lags of 0–6 years (lags 0–6, *p <* 0.0001), with the strongest associations at lags of 0 year (*z* = 8.691, *p <* 0.0001) and 1 year (*z* = 7.854, *p <* 0.0001), followed by a gradual decline with increasing delay (see Table 1).

**Table 1.** Unstandardized regression coefficients (95% CIs) for associations between THC concentration in seized cannabis resin and first-time cannabis admissions to drug treatment, at time lags of 0–10 years

Time lag(years)	*B*	SE	*Z*	*p*	95% CIlower	95% CI upper
**0**	**0.105**	**0.012**	**8.691**	**0.000**	**0.082**	**0.129**
**1**	**0.072**	**0.009**	**7.854**	**0.000**	**0.054**	**0.090**
**2**	**0.063**	**0.011**	**5.748**	**0.000**	**0.041**	**0.084**
**3**	**0.059**	**0.012**	**4.833**	**0.000**	**0.035**	**0.083**
**4**	**0.056**	**0.013**	**4.284**	**0.000**	**0.030**	**0.081**
**5**	**0.071**	**0.015**	**4.790**	**0.000**	**0.042**	**0.101**
**6**	**0.059**	**0.016**	**3.729**	**0.000**	**0.028**	**0.090**
**7**	0.043	0.018	2.369	0.018	0.007	0.078
**8**	0.047	0.021	2.181	0.029	0.005	0.088
**9**	0.006	0.023	0.266	0.790	−0.039	0.051
**10**	0.069	0.039	1.778	0.075	−0.007	0.145

*Note:* Adjusted for sex, age, and non-cannabis drug treatment admissions. Significant associations marked in bold (*p <* 0.01).

### Time-dependent associations between potency and incidence of cannabis-induced psychosis

We did not find significant associations between THC concentration and incidence of cannabis-induced psychosis when assessed in crude and in sex- and age-adjusted models. When additionally adjusting for other substance-induced psychosis, THC was positively associated with incidence of cannabis-induced psychosis at lags of 0–4 years (lags 0–2, *p* < 0.0001; lags 3–4, *p* < 0.01), with the strongest association at year 0 (*z =* 5.124, *p <* 0.0001) followed by a gradual decline with increasing delay (see Table 2).

**Table 2.** Unstandardized regression coefficients (95% CIs) for associations between THC concentration in seized cannabis resin and incidence of cannabis-induced psychosis, at time lags of 0–10 years

Time lag(years)	*B*	SE	*Z*	*P*	95% CI lower	95% CI upper
**0**	**0.012**	**0.002**	**5.124**	**0.000**	**0.007**	**0.017**
**1**	**0.008**	**0.002**	**3.737**	**0.000**	**0.004**	**0.012**
**2**	**0.007**	**0.002**	**3.727**	**0.000**	**0.003**	**0.011**
**3**	**0.005**	**0.002**	**2.597**	**0.009**	**0.001**	**0.009**
**4**	**0.006**	**0.002**	**2.606**	**0.009**	**0.002**	**0.011**
**5**	0.005	0.002	2.260	0.024	0.001	0.010
**6**	0.006	0.003	1.895	0.058	0.000	0.011
**7**	0.005	0.003	1.589	0.112	−0.001	0.012
**8**	0.003	0.003	0.802	0.423	−0.004	0.010
**9**	0.002	0.006	0.375	0.708	−0.009	0.013
**10**	−0.008	0.008	−0.953	0.340	−0.024	0.008

*Note:* Adjusted for sex, age, and other substance-induced psychosis. Significant associations marked in bold (*p <* 0.01).

### Time-dependent associations between potency and incidence of dual diagnosis

We did not find significant associations between THC concentration and incidence of dual diagnosis when assessed in crude and in sex- and age-adjusted models. When additionally adjusting for other substance use disorders, THC was positively associated with the incidence of dual diagnosis at lags of 0–1 years (lags 0–1, *p <* 0.01), with the strongest association at year 0 (*z =* 3.051, *p <* 0.01) (see Table 3).

**Table 3.** Unstandardized regression coefficients (95% CIs) for associations between THC concentration in seized cannabis resin and incidence of dual diagnosis (cannabis use disorder and schizophrenia), at time lags of 0–10 years

Time lag(years)	*B*	SE	*Z*	*P*	95% CI lower	95% CI upper
**0**	**0.006**	**0.002**	**3.051**	**0.002**	**0.002**	**0.010**
**1**	**0.005**	**0.002**	**2.673**	**0.008**	**0.001**	**0.008**
**2**	0.002	0.002	0.884	0.377	−0.002	0.005
**3**	0.001	0.002	0.388	0.698	−0.003	0.005
**4**	0.002	0.002	0.694	0.488	−0.003	0.006
**5**	0.003	0.003	1.068	0.286	−0.002	0.008
**6**	0.001	0.003	0.191	0.849	−0.005	0.006
**7**	0.001	0.003	0.276	0.783	−0.005	0.006
**8**	0.000	0.003	−0.046	0.963	−0.006	0.006
**9**	−0.004	0.005	−0.931	0.352	−0.013	0.005
**10**	−0.016	0.008	−1.951	0.051	−0.032	0.000

*Note:* Adjusted for sex, age, and other substance use disorder. Significant associations marked in bold (*p <* 0.01).

### Role of age and sex

Findings from fully adjusted models showed that THC concentration was positively associated with admission for cannabis treatment for adults (≥ 25 years), but not younger individuals (≤ 24 years), at lags of 0–1 years (strongest at year 0, *Z =* 4.77). Significant associations were found for women, but not men, at lags of 0–1 years (strongest at year 0, *Z =* 4.72, see Supplementary Tables 1A and B). Similarly, findings from fully adjusted models showed that THC concentration was positively associated with incidence of cannabis-induced psychosis among adults (≥ 25 years), but not younger individuals (≤ 24 years), at lags of 0–2 years (strongest at year 0, *Z =* 4.18); and in women, but not men, at lags of 0–1 (strongest at year 0, *Z =* 4.33, see Supplementary Tables 2A and B). We found no associations between THC concentration and incidence of dual diagnosis in subgroup analyses (see Supplementary Tables 3A and B).

### Alternative explanation: changes in the prevalence of cannabis use

**Figure 2.**
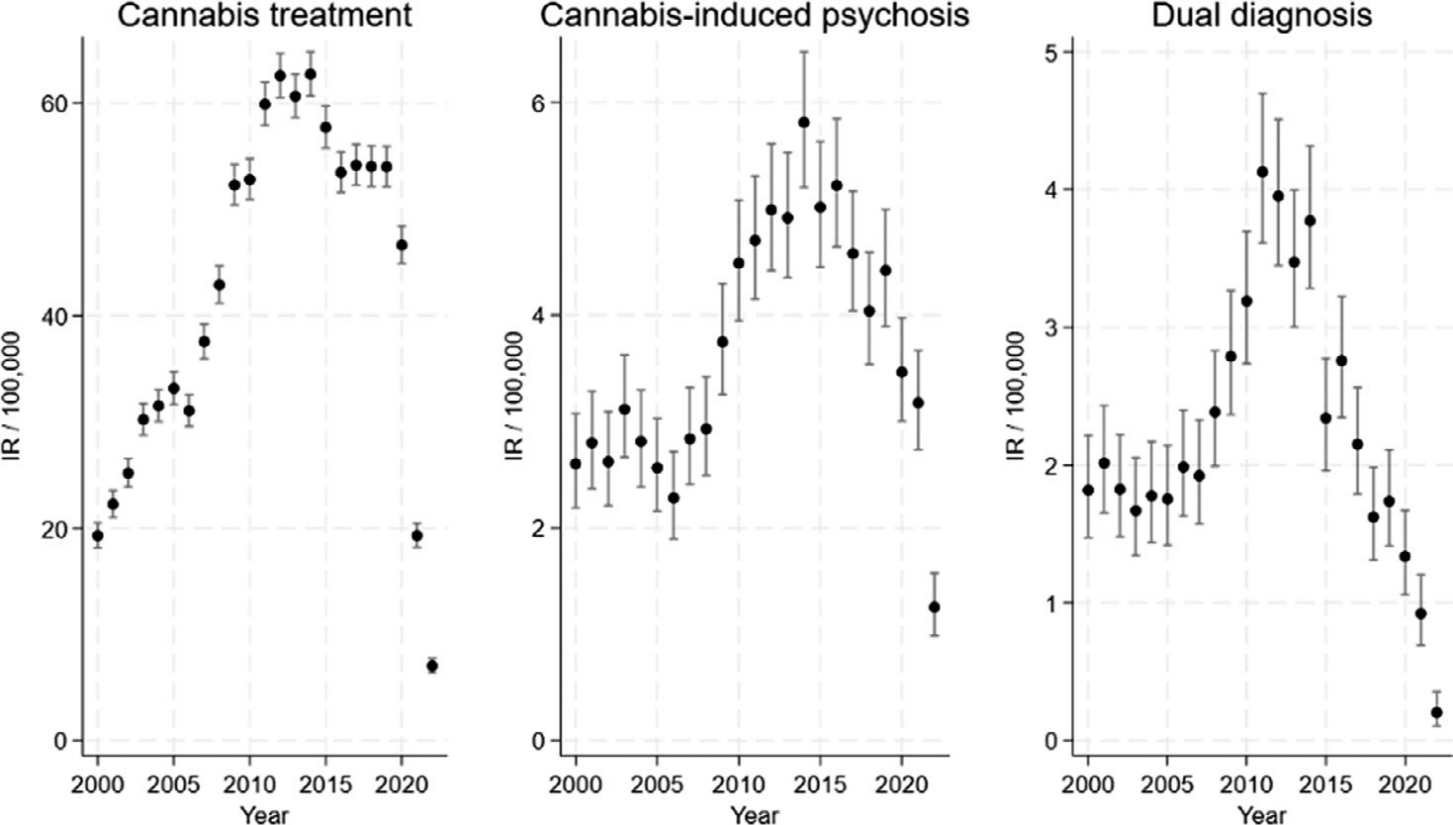
Development in outcomes over time (unadjusted).

The reported changes in the incidence rate of first-time admissions to specialist treatment for cannabis problems, cannabis-induced psychosis, and dual diagnosis, could also be attributable to other factors, such as changes in the number of people who use cannabis resin, increased use of other types of cannabis in Denmark, or changes in frequency and/or quantity of use (e.g. standard THC units), as previous studies point to the importance of frequency (Groening et al., 2024) and quantity (Lees Thorne et al., 2026), including evidence that frequency moderates the association between cannabis use and psychosis related outcomes (Groening et al., 2024). Hence, it is important to examine the development in self-reported use of cannabis resin in the period of interest (2000–2022): Data from ESPAD show fluctuations in past month and lifetime use of cannabis resin among young adolescents (15- to 16-year-olds) from 1999 to 2007, followed by a decrease (ESPAD Group, 2025) (Supplementary Table 4A). Representative national surveys conducted by the Danish Health Authority (Danish Health Authority, 2024b) show fluctuations in past month and lifetime use of cannabis resin among 16- to 24-year-olds from 2000 to 2017 and decreased use in 2021 (Supplementary Table 4B); and similarly among 16- to 44-year-olds, although the decrease in 2021 was smaller (Supplementary Table 4C). Further, representative national surveys of 15- to 25-year-olds conducted by the Centre for Alcohol and Drug Research at Aarhus University (Pedersen et al., 2023) show a stable pattern in past month use of cannabis (cannabis resin, pot, and skunk) from 2014 to 2022, and a stable pattern in frequency of use in this period (Supplementary Table 4D and E), but a decreased lifetime prevalence in 2022. Taken together, the development in patterns of self-reported use does not follow the development in the three outcomes (see Figure 2) and hence is unlikely to explain the reported increases across the three outcomes. However, the decrease in self-reported use in 2021/2022 may have contributed to the decrease across outcomes, and both (the decrease in self-reported use and the three outcomes) could be related to the COVID-19 pandemic, although findings regarding the impact of COVID-19 are mixed (Bonnet, Specka, Roser, & Scherbaum, 2023; Chong, Acar, West, & Wong, 2022).

## Discussion

In terms of the three aims of our study, we found that in the population as a whole, cannabis potency was associated with the incidence of treatment seeking for a cannabis problem, cannabis-induced psychosis, and dual diagnosis (i.e. schizophrenia and cannabis use disorder). This is significant from a public health perspective because it points to high-potency cannabis being associated with severe psychiatric harms and disability-adjusted life-years in the general population.

We found that the THC concentration in samples of seized cannabis resin increased almost fourfold from a mean of 8.3–31.2% from 2000 to 2022 in Denmark, in line with previous reports of increased potency of cannabis resin and herbal cannabis across Europe and the United States (European Unioin Drugs Agency, 2024; Freeman et al., 2021), and particularly high THC concentration of cannabis resin in Denmark (Freeman et al., 2019; Rømer Thomsen et al., 2019). As a comparison, a recent report from EUDA shows that in 2023, the average THC content of cannabis resin in the European Union was 23%, more than twice that of herbal cannabis, at 11% (European Union Drugs Agency, 2025).

In line with previous research (Freeman et al., 2018), we found that higher THC concentrations in the cannabis available on the market were associated with first-time admissions to specialized treatment for cannabis problems (in the fully adjusted model). In the previous study from the Netherlands, associations between THC and first-time cannabis treatment entry were strongest after 5 years, while we found the strongest associations at lags of 0 and 1 years, followed by a gradual decline with increasing delay until year 7, where it was no longer significant. Both studies support the hypothesis that increased potency is linked with increased risk of cannabis use disorder by providing evidence at time lags that are biologically plausible (based on age of first cannabis use and age of first treatment entry (European Union Drugs Agency, 2025; Freeman et al., 2018). Variations in time lags could be due to possible associations of increased THC exposure with different stages of addiction (e.g. transitions from mild to moderate cannabis use disorder, from moderate to severe cannabis use disorder, or from cannabis use disorder to treatment entry), as well as other factors such as cannabis markets, poly-drug use (Crummy, O’Neal, Baskin, & Ferguson, 2020; Liu et al., 2018), societal views and stigma, treatment seeking behavior (Kerridge et al., 2017; Olthof, Blankers, van Laar, & Goudriaan, 2022), and differences is THC potency across countries (Freeman et al., 2019).

In addition, we found that THC concentrations were associated with the incidence of cannabis-induced psychoses and the incidence of dual diagnosis, defined as the coexistence of schizophrenia and cannabis use disorder (in the fully adjusted models). This is in line with the recent study from Germany, which documented time-dependent associations between changes in potency of cannabis flower and the ratio of cannabis-related diagnoses, including cannabis-induced psychosis (Manthey et al., 2024). Consistent with these findings, we found the strongest effect at time lag 0, with a continuous decrease in the effect strength with increasing delay. Increases in cannabis-induced psychosis have also been recorded in neighboring countries, Sweden and Norway, although less is known about cannabis potency in these countries (Rognli et al., 2023). Cannabis-induced psychosis is of particular concern as it has the highest conversion rate to schizophrenia among substance-induced psychoses, with a conversion rate of 1/3 (Murrie et al., 2020).

Exploratory subgroup analyses indicated that associations were stronger among adults aged 25 years or older and in women. In contrast, associations between THC concentration and cannabis-related ICD-10 diagnoses were found to be more pronounced among men in the German study (Manthey et al., 2024). Due to small sample sizes, our findings from the subgroup analyses should be considered with caution. Studies designed to assess the role of age and sex, and with larger sample sizes, are needed to determine the role of age and sex in associations over time between cannabis potency and risk of cannabis-related psychiatric harms.

The reported changes in the incidence rate of first-time admissions to specialist treatment for cannabis problems, cannabis-induced psychosis, and dual diagnosis, could also be attributable to other factors, such as increases (and decreases) in the number of people who use cannabis resin/other types of cannabis in Denmark, or changes in frequency and/or quantity of use (e.g. standard THC units), as previous studies point to the importance of frequency (Groening et al., 2024) and quantity (Lees Thorne et al., 2026; Thorne et al., 2025). Findings from nationally representative surveys show relatively small fluctuations in past-month and lifetime use of cannabis resin from 2000 to 2013, and hence cannot account for the increases across outcomes. Also, the decrease in self-reported use from 2017 to 2021/2022 may have contributed to the decrease across outcomes, and both (the decrease in self-reported use and the three outcomes) could be related to the COVID-19 pandemic. However, nationally representative surveys may not accurately capture cannabis use in places where it is prohibited or socially disapproved, as found in a recent Swedish study (Andersson et al., 2023).

The expected associations were found in the fully adjusted models, but not in the crude and sex- and age-adjusted models. These findings suggest that other factors (than potency) impact the incidence of first-time admission to specialized treatment for a cannabis problem, cannabis-induced psychosis, and dual diagnosis, and blur associations with potency of seized cannabis resin. More specifically, admission to specialized treatment for cannabis problems and to diagnostic evaluation of cannabis-related disorders is impacted by systemic factors such as general treatment-seeking patterns, treatment capacity, diagnostic evaluation capacity, and overall substance use burdens (Kerridge et al., 2017; Olthof et al., 2022). For example, first-time admissions to specialized drug treatment *in general* are most likely impacted by these systemic factors. By adjusting for specialized treatment for non-cannabis drug problems, other factors related to treatment system activity are held constant, which revealed a positive association between the potency of cannabis resin and first-time admissions to specialized treatment for cannabis problems. Similarly, by adjusting for other substance-induced psychoses, these factors were held constant, allowing for positive associations between potency and cannabis-induced psychosis to emerge (and similarly with the dual diagnosis outcome). Importantly, the study period (2000–2022) covers the COVID-19 pandemic, which impacted treatment-seeking patterns and treatment and diagnostic evaluation capacity, for example, in person consultations switched to online in some periods of the pandemic and there was a decrease in psychiatric admissions in Denmark (Del Palacio-Gonzalez, Thylstrup, & Houborg, 2022; Grønkjær, Christensen, Kondziella, & Benros, 2025; Hansen et al., 2021), suggesting that the pandemic may account for part of the drop across outcomes in the last part of the study period (Figure 2). The fact that expected associations were only present in the fully adjusted models underscores the complexity and multitude of factors involved in psychiatric outcomes and stresses the need to adjust for general factors related to treatment system activity.

Taken together, the results of this study underscore that in Denmark, the European country showing the highest concentration of THC in cannabis resin reported to the EUDA in 2016 (Freeman et al., 2019), there was converging evidence for associations between THC concentration in seized cannabis resin samples and three key indicators of cannabis-related psychiatric harms (from 2000 to 2022). The strongest association was found for first-time admissions to specialized cannabis treatment, followed by cannabis-induced psychosis, and with stronger associations among adults (25 years or more) and women. The reported associations stress the importance of considering cannabis potency in healthcare and psychiatric settings and in public health and harm reduction guidelines.

The present study, and the studies from Germany (Manthey et al., 2024) and the Netherlands (Freeman et al., 2018), are ecological studies and hence we cannot establish causality in these studies alone. However, when triangulated with evidence from RCTs showing that higher doses of THC cause greater severity of harm (Curran et al., 2002; D’Souza et al., 2004; Hindley et al., 2020; Morrison et al., 2009), as well as epidemiological studies showing an association between cannabis potency with CUD and psychotic disorders (Lake et al., 2025; Petrilli et al., 2022), these findings suggest that policies focusing on cannabis potency can potentially play an important role in reducing harms. For example, domestic production of cannabis resin from high potency herbal cannabis may explain increases in the potency of cannabis resin reported in Europe (such as Denmark) rather than the import of cannabis resin from neighboring countries such as Morocco (Freeman et al., 2025). Such domestic production of high-potency cannabis resin could be targeted in an attempt to influence the potency of cannabis resin in European countries. By contrast, in legal cannabis markets, setting caps on the potency of cannabis products legally sold, and or taxing according to THC content, could potentially offset the harmful psychiatric effects of higher potency cannabis products (Hall, Leung, & Carlini, 2023).

### Limitations

A number of limitations should be considered. Data from registers are limited by the consistency of data collection over both time and geography. Thus, to the extent that psychiatric diagnoses have been made inconsistently or individuals seeking treatment have reported primary drug use inconsistently, our findings may also be influenced. Because cannabis resin samples from 2014 to 2022 stem from three police districts, we opted to restrict analyses to these areas. The rationale was that people who used cannabis and lived within 40 kilometers where likely to have obtained cannabis from these areas, but we acknowledge that this is an assumption. Furthermore, we cannot disentangle poly-drug use that includes cannabis from the use of cannabis with a small amount of co-use of other substances based on our data. Similarly, the cannabis seized by the police does not represent a random sample of all cannabis used by the population at risk. The cannabis used may be of higher or lower potency. Further, it may be seen as a limitation that only cannabis resin was analyzed. Cannabis resin dominates the market in Denmark, but other types are also used. A nationally representative survey from 2025 shows that, among 15- to 25-year-olds who used cannabis in the past year, 73% reported using cannabis resin, 41.2% skunk, and 16.1% marijuana (Frederiksen et al., 2025). Use of other types of cannabis or synthetic cannabinoids may influence the risk of cannabis-related psychiatric disorders. Further, because of the ecological design, we cannot infer causality between the studied factors. However, the combination of plausible mechanisms, temporal proximity, and consistency across studies (including triangulation with RCTs and epidemiological studies) strengthens support for the hypothesis of a role of cannabis potency in psychiatric harms at the population level (Shimonovich et al., 2021). While the sample is not small, we acknowledge that the subgroup analyses contained some small cells. This limits the power of our analyses to identify important differences between sex and age groups. Finally, analyses were restricted to two key psychotic outcomes (cannabis-induced psychosis, schizophrenia). Future studies are needed to examine psychotic outcomes more broadly or additional subtypes.

## Conclusion

THC levels in Danish cannabis resin have increased nearly fourfold over the past decades. When assessed on an ecological level, we found positive associations between potency and cannabis-related psychiatric harms in the fully adjusted models. Higher potency was linked to increases in first-time cannabis treatment entry, incidence of cannabis-induced psychosis, and incidence of dual diagnosis – especially among adult users and women for the first two outcomes. Our study adds to the growing body of evidence suggesting an association between high-potency cannabis use and negative psychiatric outcomes, and at the same time acknowledges the role of general systemic factors related to treatment and psychiatric evaluation. These findings stress the need to consider potency in healthcare and psychiatric settings and in public health and harm reduction guidelines. Relatedly, our study highlights the importance of monitoring changes in cannabis potency internationally and evaluating its potential impact on psychiatric harms at the population level.

## Supporting information

10.1017/S0033291726104036.sm001 Rømer Thomsen et al. supplementary material Rømer Thomsen et al. supplementary material

## Supplementary material

The supplementary material for this article can be found at http://doi.org/10.1017/S0033291726104036.
