# Supplementary material for: Changes in cannabis potency and cannabis-related psychiatric harms: a 23-year ecological study in Denmark
Source: Psychol Med. 2026 May 4;56:e124. doi: 10.1017/S0033291726104036 (PMC13161803; doi:10.1017/S0033291726104036)
Supplement: Rømer Thomsen et al. supplementary material [file S0033291726104036sup001.docx]

**Supplementary file 1**

We assumed that users living within 40 kilometres of Copenhagen, Aarhus, and Odense were highly likely to have obtained their cannabis resin from these cities or smaller towns within these police districts. Hence, we included individuals living in municipalities in these areas in our analyses. The included municipalities are listed below in Danish (before and after *The Structural Reform* in 2004, where the number of municipalities was changed from 271 to the current 98).

**Copenhagen** (in Danish: København)

Included municipalities *before* the reform: København, Frederiksberg, Helsingør, Fredensborg, Fredensborg-Humlebæk, Karlebo, Hillerød, Skævinge, Frederikssund, Skibby, Slangerup, Jægerspris, Allerød, Hørsholm, Rudersdal, Birkerød, Søllerød, Furesø, Farum, Værløse, Egedal, Stenløse, Ølstykke, Lyngby-Taarbæk, Gentofte, Gladsaxe, Herlev, Ballerup, Høje-Taastrup, Albertslund, Glostrup, Rødovre, Brøndby, Ishøj, Vallensbæk, Hvidovre, Tårnby, Dragør, Roskilde, Ramsø, Gundsø, Greve, Solrød, Køge, Skovbo, Stevns, Vallø.

Included municipalities *after* the reform: København, Frederiksberg, Helsingør, Fredensborg, Hillerød, Frederikssund, Allerød, Hørsholm, Rudersdal, Furesø, Egedal, Lyngby-Taarbæk, Gentofte, Gladsaxe, Herlev, Ballerup, Høje-Taastrup, Albertslund, Glostrup, Rødovre, Brøndby, Ishøj, Vallensbæk, Hvidovre, Tårnby, Dragør, Roskilde, Greve, Solrød, Køge, Stevns

**Aarhus**

Included municipalities *before* the reform: Aarhus, Silkeborg, Kjellerup, Gjern, Them, Skanderborg, Galten, Ry, Hørning, Horsens, Brændstrup, Gedved, Odder, Favrskov, Hvorslev, Hadsten, Hinnerup, Hammel, Randers, Nørhald, Purhus, Sønderhald, Syddjurs, Midtdjurs, Rosenholm, Rønde, Ebeltoft.

Included municipalities *after* the reform: Aarhus, Silkeborg, Skanderborg, Horsens, Odder, Favrskov, Randers, Syddjurs.

**Odense**

Included municipalities *before* the reform: Odense, Nyborg, Ullerslev, Ørbæk, Svendborg, Gudme, Egebjerg, Faaborg-Midt, Åslev, Broby, Ringe, Ryslinge, Faaborg, Assens, Vissenbjerg, Aarup, Tommerup, Glamsbjerg, Haarby, Middelfart, Nørre Aaby, Ejby, Nordfyns, Otterup, Bogense, Søndersø, Kerteminde, Munkebo, Langeskov.

Included municipalities *after* the reform: Odense, Nyborg, Svendborg, Faaborg-Midt, Assens, Middelfart, Nordfyns, Kerteminde.

**Supplementary Table 1A.**

**Unstandardized regression coefficients (95% CIs) for associations between THC concentration in seized cannabis resin and first-time cannabis admissions to drug treatment in young (up to 24 years) and older (25 years and older) patients, at time lags of 0-10 years.** Adjusted for sex and non-cannabis drug treatment admissions. Significant associations marked in bold (p<0.01).

| **Subgroup** | **Time lag (years)** | **Coefficient** | **SE** | **Z** | **P** | **95% CI**  **lower** | **95% CI**  **upper** |
| --- | --- | --- | --- | --- | --- | --- | --- |
| -24 | 0 | 0.52 | 0.35 | 1.47 | 0.14 | -0.17 | 1.20 |
| **25+** | **0** | **0.81** | **0.17** | **4.77** | **0.00** | **0.48** | **1.15** |
| -24 | 1 | 0.16 | 0.39 | 0.42 | 0.67 | -0.60 | 0.92 |
| **25+** | **1** | **0.59** | **0.19** | **3.07** | **0.00** | **0.21** | **0.97** |
| -24 | 2 | -0.15 | 0.41 | -0.36 | 0.72 | -0.96 | 0.66 |
| 25+ | 2 | 0.45 | 0.21 | 2.15 | 0.03 | 0.04 | 0.86 |
| -24 | 3 | -0.46 | 0.43 | -1.06 | 0.29 | -1.30 | 0.39 |
| 25+ | 3 | 0.29 | 0.23 | 1.27 | 0.20 | -0.16 | 0.73 |
| -24 | 4 | -0.54 | 0.47 | -1.17 | 0.24 | -1.45 | 0.37 |
| 25+ | 4 | 0.22 | 0.24 | 0.93 | 0.35 | -0.24 | 0.68 |
| -24 | 5 | -0.60 | 0.54 | -1.11 | 0.27 | -1.65 | 0.45 |
| 25+ | 5 | 0.25 | 0.26 | 0.98 | 0.33 | -0.25 | 0.75 |
| -24 | 6 | -0.70 | 0.61 | -1.14 | 0.25 | -1.89 | 0.50 |
| 25+ | 6 | 0.12 | 0.28 | 0.44 | 0.66 | -0.43 | 0.68 |
| -24 | 7 | -0.92 | 0.74 | -1.25 | 0.21 | -2.36 | 0.53 |
| 25+ | 7 | -0.11 | 0.29 | -0.39 | 0.70 | -0.69 | 0.46 |
| -24 | 8 | -0.85 | 1.04 | -0.82 | 0.41 | -2.88 | 1.18 |
| 25+ | 8 | -0.41 | 0.33 | -1.24 | 0.22 | -1.05 | 0.24 |
| -24 | 9 | -1.37 | 1.59 | -0.86 | 0.39 | -4.48 | 1.75 |
| 25+ | 9 | -0.48 | 0.47 | -1.01 | 0.31 | -1.41 | 0.45 |
| -24 | 10 | -1.65 | 2.05 | -0.80 | 0.42 | -5.67 | 2.37 |
| 25+ | 10 | -0.27 | 0.51 | -0.52 | 0.60 | -1.27 | 0.74 |

**Supplementary Table 1B.**

**Unstandardized regression coefficients (95% CIs) for associations between THC concentration in seized cannabis resin and first-time cannabis admissions to drug treatment in men and women, at time lags of 0-10 years.** Adjusted for age and non-cannabis drug treatment admissions. Significant associations marked in bold (p<0.01).

| **Subgroup** | **Time lag (years)** | **Coefficient** | **SE** | **Z** | **P** | **95% CI**  **lower** | **95% CI**  **upper** |
| --- | --- | --- | --- | --- | --- | --- | --- |
| Men | 0 | 0.82 | 0.33 | 2.47 | 0.01 | 0.17 | 1.47 |
| **Women** | **0** | **0.53** | **0.11** | **4.72** | **0.00** | **0.31** | **0.74** |
| Men | 1 | 0.47 | 0.38 | 1.23 | 0.22 | -0.28 | 1.21 |
| **Women** | **1** | **0.43** | **0.14** | **3.06** | **0.00** | **0.15** | **0.70** |
| Men | 2 | 0.14 | 0.41 | 0.33 | 0.74 | -0.67 | 0.95 |
| Women | 2 | 0.36 | 0.16 | 2.23 | 0.03 | 0.04 | 0.67 |
| Men | 3 | -0.19 | 0.44 | -0.42 | 0.67 | -1.04 | 0.67 |
| Women | 3 | 0.24 | 0.18 | 1.30 | 0.19 | -0.12 | 0.59 |
| Men | 4 | -0.26 | 0.47 | -0.54 | 0.59 | -1.18 | 0.67 |
| Women | 4 | 0.16 | 0.20 | 0.83 | 0.41 | -0.22 | 0.55 |
| Men | 5 | -0.27 | 0.54 | -0.50 | 0.62 | -1.33 | 0.79 |
| Women | 5 | 0.11 | 0.23 | 0.50 | 0.62 | -0.33 | 0.55 |
| Men | 6 | -0.43 | 0.62 | -0.69 | 0.49 | -1.64 | 0.79 |
| Women | 6 | 0.04 | 0.26 | 0.17 | 0.87 | -0.46 | 0.55 |
| Men | 7 | -0.73 | 0.76 | -0.96 | 0.34 | -2.22 | 0.76 |
| Women | 7 | -0.17 | 0.29 | -0.58 | 0.56 | -0.74 | 0.40 |
| Men | 8 | -0.87 | 1.08 | -0.81 | 0.42 | -2.98 | 1.24 |
| Women | 8 | -0.31 | 0.40 | -0.77 | 0.44 | -1.10 | 0.48 |
| Men | 9 | -1.42 | 1.60 | -0.88 | 0.38 | -4.56 | 1.73 |
| Women | 9 | -0.33 | 0.61 | -0.55 | 0.59 | -1.54 | 0.87 |
| Men | 10 | -1.60 | 1.81 | -0.89 | 0.38 | -5.16 | 1.95 |
| Women | 10 | 0.02 | 0.64 | 0.02 | 0.98 | -1.24 | 1.28 |

**Supplementary Table 2A.**

**Unstandardized regression coefficients (95% CIs) for associations between THC concentration in seized cannabis resin and incidence of cannabis-induced psychosis in young (up to 24 years) and older (25 years and older) patients, at time lags of 0-10 years.** Adjusted for sex and other substance-induced psychosis. Significant associations marked in bold (p<0.01).

| **Subgroup** | **Time lag (years)** | **Coefficient** | **SE** | **Z** | **P** | **95% CI**  **lower** | **95% CI**  **upper** |
| --- | --- | --- | --- | --- | --- | --- | --- |
| -24 | 0 | 0.03 | 0.03 | 0.93 | 0.35 | -0.03 | 0.10 |
| **25+** | **0** | **0.07** | **0.02** | **4.18** | **0.00** | **0.04** | **0.10** |
| -24 | 1 | 0.00 | 0.04 | 0.02 | 0.99 | -0.07 | 0.07 |
| **25+** | **1** | **0.07** | **0.02** | **3.77** | **0.00** | **0.03** | **0.11** |
| -24 | 2 | -0.04 | 0.04 | -1.06 | 0.29 | -0.11 | 0.03 |
| **25+** | **2** | **0.07** | **0.02** | **3.07** | **0.00** | **0.02** | **0.11** |
| -24 | 3 | -0.05 | 0.04 | -1.41 | 0.16 | -0.12 | 0.02 |
| 25+ | 3 | 0.05 | 0.02 | 1.94 | 0.05 | 0.00 | 0.09 |
| -24 | 4 | -0.04 | 0.04 | -1.01 | 0.31 | -0.12 | 0.04 |
| 25+ | 4 | 0.04 | 0.03 | 1.35 | 0.18 | -0.02 | 0.09 |
| -24 | 5 | -0.03 | 0.05 | -0.65 | 0.51 | -0.13 | 0.06 |
| 25+ | 5 | 0.04 | 0.03 | 1.20 | 0.23 | -0.02 | 0.10 |
| -24 | 6 | -0.05 | 0.06 | -0.89 | 0.37 | -0.16 | 0.06 |
| 25+ | 6 | 0.02 | 0.04 | 0.53 | 0.59 | -0.05 | 0.09 |
| -24 | 7 | -0.06 | 0.07 | -0.84 | 0.40 | -0.18 | 0.07 |
| 25+ | 7 | 0.00 | 0.04 | -0.06 | 0.95 | -0.08 | 0.07 |
| -24 | 8 | -0.09 | 0.09 | -1.05 | 0.29 | -0.26 | 0.08 |
| 25+ | 8 | -0.03 | 0.04 | -0.85 | 0.39 | -0.12 | 0.05 |
| -24 | 9 | -0.16 | 0.11 | -1.50 | 0.13 | -0.37 | 0.05 |
| 25+ | 9 | -0.05 | 0.06 | -0.87 | 0.38 | -0.16 | 0.06 |
| -24 | 10 | -0.30 | 0.16 | -1.95 | 0.05 | -0.61 | 0.00 |
| 25+ | 10 | -0.03 | 0.08 | -0.39 | 0.69 | -0.19 | 0.13 |

**Supplementary Table 2B.**

**Unstandardized regression coefficients (95% CIs) for associations between THC concentration in seized cannabis resin and incidence of cannabis-induced psychosis in men and women, at time lags of 0-10 years.** Adjusted for sex and other substance-induced psychosis. Significant associations marked in bold (p<0.01).

| **Subgroup** | **Time lag (years)** | **Coefficient** | **SE** | **Z** | **P** | **95% CI**  **lower** | **95% CI**  **upper** |
| --- | --- | --- | --- | --- | --- | --- | --- |
| Men | 0 | 0.07 | 0.04 | 2.07 | 0.04 | 0.00 | 0.14 |
| **Women** | **0** | **0.04** | **0.01** | **4.33** | **0.00** | **0.02** | **0.06** |
| Men | 1 | 0.05 | 0.04 | 1.16 | 0.25 | -0.03 | 0.12 |
| **Women** | **1** | **0.04** | **0.01** | **3.35** | **0.00** | **0.02** | **0.06** |
| Men | 2 | 0.01 | 0.04 | 0.21 | 0.83 | -0.07 | 0.09 |
| Women | 2 | 0.03 | 0.01 | 2.79 | 0.01 | 0.01 | 0.06 |
| Men | 3 | -0.02 | 0.04 | -0.48 | 0.63 | -0.11 | 0.07 |
| Women | 3 | 0.03 | 0.01 | 1.91 | 0.06 | 0.00 | 0.05 |
| Men | 4 | -0.02 | 0.05 | -0.41 | 0.68 | -0.12 | 0.08 |
| Women | 4 | 0.02 | 0.02 | 1.27 | 0.20 | -0.01 | 0.05 |
| Men | 5 | -0.01 | 0.06 | -0.12 | 0.90 | -0.12 | 0.11 |
| Women | 5 | 0.01 | 0.02 | 0.77 | 0.44 | -0.02 | 0.05 |
| Men | 6 | -0.04 | 0.07 | -0.57 | 0.57 | -0.17 | 0.09 |
| Women | 6 | 0.00 | 0.02 | -0.18 | 0.86 | -0.04 | 0.04 |
| Men | 7 | -0.05 | 0.09 | -0.57 | 0.57 | -0.22 | 0.12 |
| Women | 7 | -0.02 | 0.02 | -1.00 | 0.32 | -0.06 | 0.02 |
| Men | 8 | -0.13 | 0.11 | -1.11 | 0.26 | -0.35 | 0.10 |
| Women | 8 | -0.04 | 0.02 | -1.71 | 0.09 | -0.09 | 0.01 |
| Men | 9 | -0.18 | 0.13 | -1.36 | 0.17 | -0.43 | 0.08 |
| Women | 9 | -0.06 | 0.03 | -1.98 | 0.05 | -0.11 | 0.00 |
| Men | 10 | -0.28 | 0.18 | -1.59 | 0.11 | -0.63 | 0.07 |
| Women | 10 | -0.08 | 0.04 | -1.76 | 0.08 | -0.16 | 0.01 |

**Supplementary Table 3A.**

**Unstandardized regression coefficients (95% CIs) for associations between THC concentration in seized cannabis resin and incidence of dual diagnosis (cannabis use disorder and schizophrenia) in young (up to 24 years) and older (25 years and older) patients, at time lags of 0-10 years.** Adjusted for sex and other substance use disorder.

| **Subgroup** | **Time lag (years)** | **Coefficient** | **SE** | **Z** | **P** | **95% CI**  **lower** | **95% CI**  **upper** |
| --- | --- | --- | --- | --- | --- | --- | --- |
| -24 | 0 | 0.05 | 0.02 | 2.34 | 0.02 | 0.01 | 0.08 |
| 25+ | 0 | 0.00 | 0.01 | 0.55 | 0.59 | -0.01 | 0.02 |
| -24 | 1 | 0.04 | 0.02 | 1.63 | 0.10 | -0.01 | 0.08 |
| 25+ | 1 | 0.00 | 0.01 | -0.13 | 0.89 | -0.02 | 0.02 |
| -24 | 2 | 0.02 | 0.02 | 0.91 | 0.36 | -0.03 | 0.07 |
| 25+ | 2 | 0.00 | 0.01 | 0.03 | 0.98 | -0.02 | 0.02 |
| -24 | 3 | 0.00 | 0.03 | 0.17 | 0.86 | -0.05 | 0.06 |
| 25+ | 3 | -0.02 | 0.01 | -1.49 | 0.14 | -0.04 | 0.01 |
| -24 | 4 | 0.00 | 0.03 | -0.04 | 0.97 | -0.06 | 0.06 |
| 25+ | 4 | -0.02 | 0.01 | -1.65 | 0.10 | -0.05 | 0.00 |
| -24 | 5 | 0.02 | 0.03 | 0.52 | 0.60 | -0.05 | 0.09 |
| 25+ | 5 | -0.03 | 0.01 | -1.83 | 0.07 | -0.05 | 0.00 |
| -24 | 6 | 0.01 | 0.04 | 0.38 | 0.70 | -0.06 | 0.09 |
| 25+ | 6 | -0.03 | 0.02 | -1.69 | 0.09 | -0.06 | 0.00 |
| -24 | 7 | 0.04 | 0.04 | 1.02 | 0.31 | -0.04 | 0.12 |
| 25+ | 7 | -0.03 | 0.02 | -1.61 | 0.11 | -0.06 | 0.01 |
| -24 | 8 | 0.07 | 0.05 | 1.26 | 0.21 | -0.04 | 0.18 |
| 25+ | 8 | -0.02 | 0.03 | -0.85 | 0.40 | -0.07 | 0.03 |
| -24 | 9 | 0.13 | 0.08 | 1.58 | 0.11 | -0.03 | 0.30 |
| 25+ | 9 | -0.03 | 0.04 | -0.73 | 0.47 | -0.11 | 0.05 |
| -24 | 10 | -0.02 | 0.11 | -0.15 | 0.88 | -0.23 | 0.20 |
| 25+ | 10 | 0.01 | 0.06 | 0.11 | 0.92 | -0.11 | 0.12 |

**Supplementary Table 3B.**

**Unstandardized regression coefficients (95% CIs) for associations between THC concentration in seized cannabis resin and incidence of dual diagnosis (cannabis use disorder and schizophrenia) in men and women, at time lags of 0-10 years.** Adjusted for age and other substance use disorder.

| **Subgroup** | **Time lag (years)** | **Coefficient** | **SE** | **Z** | **P** | **95% CI**  **lower** | **95% CI**  **upper** |
| --- | --- | --- | --- | --- | --- | --- | --- |
| Men | 0 | 0.04 | 0.02 | 1.69 | 0.09 | -0.01 | 0.08 |
| Women | 0 | 0.02 | 0.01 | 1.97 | 0.05 | 0.00 | 0.04 |
| Men | 1 | 0.02 | 0.03 | 0.83 | 0.41 | -0.03 | 0.07 |
| Women | 1 | 0.02 | 0.01 | 1.69 | 0.09 | 0.00 | 0.04 |
| Men | 2 | 0.01 | 0.03 | 0.40 | 0.69 | -0.05 | 0.07 |
| Women | 2 | 0.01 | 0.01 | 1.42 | 0.16 | -0.01 | 0.03 |
| Men | 3 | -0.01 | 0.03 | -0.17 | 0.86 | -0.07 | 0.06 |
| Women | 3 | 0.00 | 0.01 | -0.05 | 0.96 | -0.02 | 0.02 |
| Men | 4 | -0.01 | 0.04 | -0.29 | 0.77 | -0.08 | 0.06 |
| Women | 4 | -0.01 | 0.01 | -0.73 | 0.47 | -0.03 | 0.01 |
| Men | 5 | 0.01 | 0.04 | 0.25 | 0.81 | -0.07 | 0.10 |
| Women | 5 | -0.01 | 0.01 | -0.99 | 0.32 | -0.03 | 0.01 |
| Men | 6 | 0.00 | 0.04 | 0.00 | 1.00 | -0.09 | 0.09 |
| Women | 6 | -0.02 | 0.01 | -1.18 | 0.24 | -0.04 | 0.01 |
| Men | 7 | 0.02 | 0.04 | 0.50 | 0.62 | -0.07 | 0.11 |
| Women | 7 | -0.02 | 0.02 | -1.57 | 0.12 | -0.06 | 0.01 |
| Men | 8 | 0.05 | 0.06 | 0.83 | 0.41 | -0.06 | 0.16 |
| Women | 8 | -0.05 | 0.02 | -2.36 | 0.02 | -0.10 | -0.01 |
| Men | 9 | 0.07 | 0.09 | 0.82 | 0.41 | -0.10 | 0.24 |
| Women | 9 | -0.07 | 0.04 | -1.79 | 0.07 | -0.15 | 0.01 |
| Men | 10 | -0.07 | 0.10 | -0.63 | 0.53 | -0.27 | 0.14 |
| Women | 10 | -0.03 | 0.06 | -0.58 | 0.56 | -0.15 | 0.08 |

**Supplementary Table 4.**

**Self-reported use of cannabis resin/cannabis from representative national surveys in Denmark**

**A. Use of cannabis resin: 15-16-year-olds**

| **Year** | **1999**  (n=1548) | **2003**  (n=2519) | **2007**  (n=881) | **2011**  (n=2190) | **2015***  (n=1670) | **2019***  (n=2488) | **2024** |
| --- | --- | --- | --- | --- | --- | --- | --- |
| Past month (%) | 7.9 | 7.2 | 10.3 | 6 | 5 | 7.4 | 3.5 |
| Lifetime (%) | 24 | 22.2 | 25.5 | 18 | 12.5 | 17 | 12 |

Data from ESPAD (ESPAD Group, 2025). In 2015 and 2019 items are related to “cannabis resin, marijuana and pot”, and not just “cannabis resin”.

**B. Use of cannabis resin: 16-24-year-olds**

| **Year** | **2000**  (n=1728) | **2005**  (n=919) | **2008**  (n=862) | **2010**  (n=1643) | **2013**  (n=1652) | **2017**  (n=1392) | **2021**  (n=1015) | **2023**  (n=766) |
| --- | --- | --- | --- | --- | --- | --- | --- | --- |
| Past month (%) | 7.8 | 7.9 | 8.1 | 8.1 | 9.5 | 8.9 | 5.8 | 7 |
| Lifetime (%) | 42.5 | 44.4 | 41.1 | 40.6 | 42 | 42.8 | 32.5 | 31.9 |

Based on data from The Danish Health Data Authority (Danish Health Authority, 2024). Items only include cannabis resin.

**C. Use of cannabis resin: 16-44-year-olds**

| **Year** | **2000**  (n=6878) | **2005**  (n=4440) | **2008**  (n=2219) | **2010**  (n=5748) | **2013**  (n=5013) | **2017**  (n=4571) | **2021**  (n=3111) | **2023**  (n=2626) |
| --- | --- | --- | --- | --- | --- | --- | --- | --- |
| Past month (%) | 4.6 | 4.2 | 3.5 | 4.2 | 5.3 | 5.5 | 3.9 | 4.8 |
| Lifetime (%) | 43.6 | 46.6 | 45.1 | 43.4 | 45.4 | 45.4 | 42.9 | 42.6 |

Based on data from The Danish Health Data Authority (Danish Health Authority, 2024). Items only include cannabis resin.

**D. Use of cannabis: 15-25-year-olds**

| **Year** | **2014**  (n=2878) | **2019**  (n=2856) | **2022**  (n=1929) |
| --- | --- | --- | --- |
| Past month (%) | 10.5 | 9.4 | 9.7 |
| Lifetime (%) | 44.0 | 44.0 | 35.7 |

Based on data from Centre for Alcohol and Drug Research, Aarhus University. Items include cannabis resin, pot, and skunk (Pedersen et al., 2023).

**E. Frequency of cannabis use (past month): 15-25-year-olds**

| **Year** | **2014**  (n=2856) | **2019**  (n=2836) | **2022**  (n=1856) |
| --- | --- | --- | --- |
| 0 days (%) | 89.5 | 90.6 | 90.2 |
| 1-3 days (%) | 5.6 | 5.2 | 5.2 |
| 4-9 days (%) | 1.8 | 1.0 | 1.2 |
| 10-19 days (%) | 1.1 | 1.2 | 1.4 |
| 20+ days (%) | 2.1 | 2.0 | 2.0 |

Based on data from Centre for Alcohol and Drug Research, Aarhus University. Items include cannabis resin, pot, and skunk (Pedersen et al., 2023).

**References**

Danish Health Authority. (2024). *Udbredelse af illegale stoffer i befolkningen og blandt de unge. Narkotikasituationen i Danmark 2024 - delrapport 1 [Prevalence of illegal drugs in the population and among youth. The Drug Situation in Denmark 2024 - sub-report 1]*.

ESPAD Group. (2025). *Key findings from the 2024 European School Survey Project on Alcohol and Other Drugs (ESPAD)*. European Union Drugs Agency. <https://doi.org/10.2810/5746644>

Pedersen, M. U., Karsberg, S. H., Pedersen, M. M., Skov, K. B. E., Frederiksen, K. S., & Fabricius, V. A. V. (2023). *Danske unges brug af rusmidler 2022. Hverdagsfunktion, mistrivsel og traumeoplevelser [Substanse use among youth in Denmark 2022]*.
